# Hypocapnia, eucapnia, and hypercapnia during “Where’s Waldo” search paradigms: Neurovascular coupling across the cardiac cycle and biological sexes

**DOI:** 10.1177/0271678X251318922

**Published:** 2025-02-04

**Authors:** Nathan E Johnson, Joel S Burma, Matthew G Neill, Joshua J Burkart, Elizabeth KS Fletcher, Jonathan D Smirl

**Affiliations:** 1 Cerebrovascular Concussion Lab, Faculty of Kinesiology, 2129 University of Calgary, Alberta, Canada; 2 Sport Injury Prevention Research Centre, Faculty of Kinesiology, 2129 University of Calgary, Calgary, Alberta, Canada; 3 Human Performance Laboratory, Faculty of Kinesiology, 2129 University of Calgary, Calgary, Alberta, Canada; 4 Libin Cardiovascular Institute of Alberta, University of Calgary, Alberta, Canada; 5 Alberta Children’s Hospital Research Institute, University of Calgary, Calgary, Alberta, Canada; 6 Hotchkiss Brain Institute, University of Calgary, Calgary, Alberta, Canada; 7 Integrated Concussion Research Program, University of Calgary, Calgary, AB, Canada

**Keywords:** Biological sex differences, cerebral blood velocity, hypercapnia, hypocapnia, neurovascular coupling

## Abstract

This investigation explored the impact of partial pressure of end-tidal carbon dioxide (P_ET_CO_2_) alterations on temporal neurovascular coupling (NVC) responses across the cardiac cycle and the influence of biological sex via a complex visual scene-search task (“*Where’s Waldo?*”). 10 females and 10 males completed five puzzles, each with 40 seconds of eyes open and 20 seconds of eyes closed, under P_ET_CO_2_ clamped at ∼40 mmHg (eucapnia), ∼55 mmHg (hypercapnia), and ∼25 mmHg (hypocapnia). Cerebral blood velocity (CBv) in the middle and posterior cerebral arteries (MCAv, PCAv) were measured via Transcranial Doppler ultrasound. Linear mixed-effects models with participants as a random effect analyzed NVC metrics, including baseline and peak CBv, relative increase, and area-under-the-curve (AUC30). During hypercapnic trials, reductions in PCAv and MCAv AUC30 were noted across the cardiac cycle (all *p < 0.001*). Hypocapnic PCAv AUC30 was reduced (all *p < 0.012*), as was systolic MCAv AUC30 (*p = 0.003*). Females displayed greater baseline PCA diastole (*p = 0.048*). No other biological sex differences were observed across conditions in baseline (all *p > 0.050*), peak (all *p* > *0.054*), relative increase (all *p > 0.511*), and AUC30 metrics (all *p > 0.514*). Despite differences in responses to hypercapnic and hypocapnic stimuli, NVC responses to complex visual tasks remain robust, across the physiological CO_2_ range.

## Introduction

Despite comprising only 2% of the body’s overall weight, the human brain is remarkably intricate and utilizes 20% of total oxygen metabolism at rest.^1–3^ Due to the limited ability to store vital substances, the brain relies heavily on multiple complex regulatory processes to ensure sufficient cerebral blood flow (CBF) to maintain neural processing. Neurovascular coupling (NVC), also referred to as functional hyperemia,^4^ describes the relationship between neurons and vasculature, their role in enhancing blood supply in active regions of the brain, and the removal of metabolic by-products produced by increased activity.^5^ NVC plays a critical role in supplying the active cortical areas of the brain with sufficient CBF to support neural activities.^5^ Moreover, NVC dysfunction is associated with many clinical conditions, including Alzheimer’s disease,^6^ spinal cord injury,^7^ and traumatic brain injuries.^8,9^

A variety of stimuli have been used to evoke an NVC response, including cognitive-based tasks,^10,11^ motor-based tasks,^12,13^ and visual-based tasks.^14–16^ Visual-based tasks that elicit NVC responses include viewing simple shapes and checkerboards, reading articles, and visual scene searches.^16^ A complex visual scene search paradigm such as *“Where’s Waldo?”*, demonstrates the greatest total haemodynamic response (i.e., area-under-the-curve) and an enhanced signal-to-noise ratio.^14,16^ Moreover, Transcranial Doppler ultrasound (TCD) is a well-established method to measure NVC responses across various tasks due to its superior temporal resolution compared to other imaging techniques such as magnetic resonance imaging, spectroscopy, and tomography.^16–18^

Cerebrovascular reactivity (CVR) represents another key regulatory process employed by the brain and describes the ability of the cerebrovasculature to respond to vasoactive stimuli.^19^ Brain blood vessels are highly responsive to changes in carbon dioxide (CO_2_) levels in the blood, with hypercapnia and hypocapnia causing significant vasodilation and vasoconstriction responses, respectively.^19–21^ Previous research has shown the efficacy of dynamic end-tidal forcing (DEF) to manipulate end-tidal gas levels (and in turn, arterial levels) by utilizing feedback and feedforward algorithms.^22,23^ Specifically, DEF can precisely adjust the end-tidal pressure of CO_2_ (P_ET_CO_2_) and oxygen (P_ET_O_2_) by altering the fraction of inspired CO_2_ and oxygen on a breath-by-breath basis.^23^

Previous research investigating the influence of CO_2_ tensions on the NVC response has yielded mixed results. Under hypercapnic conditions, one study reported an attenuated NVC response during a passive motor task.^24^ Another observed no changes in NVC response during a reading visual paradigm.^25^ During hypocapnic challenges, NVC response is reported to decrease during a visual simulation,^26^ while others have reported no changes in total activation.^27^ Collectively, these studies only describe data measured from the mean aspect of the cardiac cycle. Therefore, further research is warranted assessing the NVC response during diastole and systole.

Additionally, there is a lack of research highlighting differences in NVC responses between biological sexes concerning CO_2_ tensions. Under eucapnic conditions, sex differences in the NVC response have been observed in baseline and peak cerebral blood velocity (CBv) metrics, with the diastolic phases of the cardiac cycle demonstrating the most apparent differences.^28^ However, sex differences were not as evident in total activation or relative CBv increases.^28^ Moreover, without further studies investigating NVC responses under various CO_2_ tensions, it remains unclear if these biological sex differences observed during eucapnic trials are influenced by CO_2_.

Therefore, the current study aimed to explore the impact of CVR across three stages- hypocapnia, eucapnia, and hypercapnia- on NVC across the cardiac cycle during a *“Where’s Waldo?”* search paradigm. Secondarily, the study sought to understand how NVC responses differ between biological sexes across cardiac cycle phases and the physiological CO_2_ spectrum. It was hypothesized that across CO_2_ tensions, females would display greater baseline and peak CBv across the cardiac cycle, as this biological sex difference has been noted under eucapnic conditions.^28,29^ Based on previous studies, it was also hypothesized both hyper- and hypocapnia would attenuate NVC,^24–26^ demonstrated by a reduction in relative increase and reduced total activation associated with the NVC stimuli.

## Materials and methods

### Ethical approval

The University of Calgary Conjoint Health Research Ethics Board (REB-20-1662 and REB 20-2112) provided approval for the current investigation. Before data collection, participants received detailed explanations about the protocol and the equipment used. Participants provided written informed consent before the start of data collection. All procedures followed institutional guidelines and adhered to the principles outlined in the Declaration of Helsinki,^30^ except for study registration within a database.

### Participants and study design

A convenience sample of 20 healthy adults (10 female, 10 male) aged 19–34, participated in a single laboratory session. Participants provided information about both their biological sex and their gender identity, with all indicating cis-gender identity. Accordingly, data will be analyzed for differences associated with biological sex, as the sample is not powered to consider the societal influences of gender. However, there is a recognized need for future research involving non-cis-gendered individuals to enhance and broaden understanding in the cerebrovascular field regarding the influence of gender on related metrics.^31,32^

The average age of female participants was 21.6 ± 1.1 years, with a body mass index (BMI) of 25.4 ± 4.2 kg/m^2^. Male participants were 25.1 ± 4.3 years of age, on average and had a BMI of 24.1 ± 2.0 kg/m^2^. Exclusion criteria included individuals with cardiorespiratory, musculoskeletal, neurological, and/or cerebrovascular conditions, or who have sustained a concussion within the preceding six months.^33^

On the day of the study, participants were instructed to abstain from consuming caffeine, alcohol, smoking, or vaping for at least 8 hours before data collection.^34–37^ Additionally, they were advised to refrain from engaging in exercise for 6 hours prior to testing.^38^

### Experimental protocols

The study was conducted at the Cerebrovascular Concussion Laboratory, University of Calgary, situated at an elevation of 1,111 meters above sea level. Upon obtaining written consent, the participant’s biological sex, gender, age, height, and weight were recorded. Subsequently, participants underwent a detailed repeated-measures protocol within the single visit to the lab, which included both an NVC task utilizing a *“Where’s Waldo”* search paradigm, alongside a dynamic cerebral autoregulation challenge using repeated squat-stand maneuvers (SSM) at two separate frequencies (0.05 Hz and 0.10 Hz) as previously described.^39–44^ The results of the cerebral autoregulation task will be published elsewhere.

The protocol was informed by the recent consensus review on NVC assessment^45^ although with the inclusion of 20 second eye-closed periods rather than 30-second periods, which is consistent with previous literature employing similar protocols.^14,16,28^ Participants were seated approximately 50–60 cm from a 27-inch monitor set to maximum brightness.^45^ Furthermore, participants wore corrective contacts or glasses if necessary to ensure 20/20 vision, eliminating the effects of nearsightedness or farsightedness. To reduce the potential confounding of a fluctuating physiological measures such as blood pressure during a single round of the task, participants completed five rounds of the *“Where’s Waldo?”* task.^5,46^ Each round consisted of 20 seconds with eyes closed (baseline), followed by 40 seconds of eyes open (activation), during which participants search for “*Waldo*” and other characters within the “*Waldo Universe”*, including “*Wenda*”, “*Odlaw*”, “*Wizard Whitebeard*”, and “*Woof’s tail*”.^45,47^ To maintain their engagement, a different puzzle was presented for each round. None of the participants managed to accomplish the task of finding all of the characters in the *“Waldo Universe”* within the allotted 40-second period, minimizing the potential influence of task-related engagement/motivation.^14^

Throughout the testing session, the barometric pressure was measured to be 666.4 ± 4.4 mmHg, the humidity.^16^ 8 ± 6.9%, and the room temperature 22.0 ± 0.7 °C. The testing commenced with the eucapnic breathing challenge, during which P_ET_CO_2_ levels were maintained at 40 mmHg. Participants completed 5 minutes of the *“Where’s Waldo?*” search paradigm, with each puzzle consisting of 40 seconds of eyes open and 20 seconds of eyes closed per puzzle.^16,28^ This protocol was then repeated at hypercapnic levels (P_ET_CO_2_ clamped at 55 mmHg) and hypocapnic levels (P_ET_CO_2_ clamped at 25 mmHg).^48^ The selection of these P_ET_CO_2_ stages were based on previous literature indicating their efficacy in inducing hypocapnia and hypercapnia at low altitudes.^23,48^ Moreover, the order of conditions (eucapnia, hypercapnia, and hypocapnia) was selected based on previous research suggesting vasoconstriction responses are less affected by moderate-to-high intensity exercise, which induces vasodilation similar to the hypercapnic condition.^49^ Furthermore, a “washout” period of 5 minutes was provided between the hypercapnic and hypocapnic stages to allow P_ET_CO_2_ to return to baseline.^49^ Each participant underwent all three conditions of the protocol during the same visit, acting as their own eucapnic controls for the hypercapnic and hypocapnic stages. This approach helps to reduce the influence of confounding factors when comparing tasks such as the influence of the menstrual cycle phase or cardiac fitness status.^50^ The total duration of the single-visit testing session was approximately 1.5 hours.

### Instrumentation

During the data collection setup, participants’ middle cerebral artery (MCA) and posterior cerebral artery (PCA) were unilaterally insonated using TCD (DWL USA, Inc, San Juan Capistrano, CA, USA) to measure CBv as a surrogate for CBF.^51^ Two 2-MHz ultrasound probes were positioned at the transtemporal window to insonate the P1 and M2 segments of the left PCA and right MCA, respectively. Trained sonographers utilized carotid compressions and simple visual tasks to confirm correct vessel insonation.^52^ Subsequently, the TCD headframe was utilized to ensure proper probe placement and maintain position throughout the testing period (DWL USA, Inc, San Juan Capistrano, CA, USA). Blood pressure (BP) was measured on a beat-to-beat basis, and pulsatile waveforms were recorded using a finger photoplethysmography device with a height correction unit (Finometer NOVA; Finapres Medical Systems, Amsterdam, The Netherlands).

Throughout the protocol, a portable DEF system, as described by Tymko et al.,^23^ was employed to regulate P_ET_CO_2_ and P_ET_O_2_ levels on a breath-by-breath basis. This system utilizes independent solenoid valves for nitrogen, oxygen, and carbon dioxide to ensure precise control over P_ET_CO_2_ and P_ET_O_2_ metrics through feedback and feedforward mechanisms.^23^ End-tidal steady state was achieved when P_ET_CO_2_ was within 1 mmHg of the target stage for three consecutive breaths.^23^ The DEF system offers advantages such as accurately maintaining P_ET_CO_2_ levels at desired values and enhancing control through adjustments in the volume injected into the inspiratory reserve via the aforementioned feedback and feedforward mechanisms.^22,23^

Participants wore a nose clip while breathing into a mouthpiece connected to a sampling line that fed into the gas analyzer (ML206; AD Instruments, Colorado Springs, CO, USA). All data were simultaneously sampled at 1000 Hz using an analog-to-digital conversion (PowerLab 16/30 ML880; ADInstruments, Colorado Springs, CO, USA) and stored using commercially available software for analysis (LabChart Pro Version 8 AD Instruments).

### Data processing

Beat-to-beat systolic and diastolic values were calculated based on the raw BP, MCA, and PCA readings. Using the precise timing of CBv and recordings from TCD and the finger photoplethysmography device, average values for these variables were obtained by averaging data points from each heartbeat. Peak levels of P_ET_CO_2_ were assessed on a breath-by-breath basis. Less than 0.5% of the data showed artifacts, which were corrected using a median filter in LabChart. Most artifacts were observed in the systolic phase of CBv recordings.

Each participant’s fifteen trials (5 hypocapnic, 5 eucapnic, and 5 hypercapnic) were synchronized with the moment-in-time eyes opened. As described previously, the analysis focused on the 5 seconds before and 30 seconds after this event.^28^ Specifically, the measures included: 1) baseline CBv averaged over 5 seconds before eye-opening, 2) highest CBv during the initial 30 seconds of task engagement, 3) Relative percentage increase in CBv from baseline to peak, and 4) Area under the curve during 30 seconds of task engagement (AUC30).^28^ AUC30 was calculated as the area under the curve for the CBV response over the first 30 seconds of the eyes-open period, using the baseline CBv calculated. This metrics captures the magnitude and duration of vascular activation, which can differentiate NVC responses based on sustained or attenuated CBv patterns over time.^16^ The AUC30 metric provides complimentary insight into subtle vasculature changes that may not be reflected by the mean response, offering additional sensitivity to transient vascular dynamics. Moreover, a strength of the current investigation in using AUC30 is that it relies on absolute CBv changes, compared to relative changes, leading to more robust comparison based on different CBv starting points between conditions (eucapnia, hypocapnia, and hypercapnia). Cleaned data were used to compute the previously mentioned metrics for the diastolic, mean, and systolic phases of the cardiac cycle in both the PCA and MCA via custom Excel scripts.

### Sample size calculation

To determine the required sample size for the study, G*Power (Version 3.1.9.6) was utilized to conduct an *a priori* power analysis using a multiple linear regression model. Based on previous investigations showing large changes in PCAv during eucapnic and hypocapnic NVC challenges,^27^ a large Cohen’s *f*^ 2^ effect size of 0.35 was chosen, with alpha set at 0.05, and a power of 0.80 targeted. Since the study hypotheses were directional, a one-tailed test was selected. Additionally, the regression model included two predictor variables: P_ET_CO2 condition and biological sex. Based on these parameters, the power analysis revealed a sample size of 20 participants would be necessary to detect the expected effects using a linear model. However, previous studies have determined that linear mixed-effect (LME) models increase power,^53^ therefore, this sample would be adequate for LME modeling as well. Additionally, it is worth noting there is currently no consistent agreement on the *a priori* sample size for LME models.^54^ Furthermore, a repeated measures protocol was designed to allow participants to function as their own controls for within subject factors. This approach helps mitigate the risk of confounding by unmeasured within-subject variables like prior concussion history, genetic factors, and other potential influences, which could otherwise impact the study outcomes while still permitting group level comparisons across sexes.^55^

### Statistical analysis

The statistical analyses were conducted using R-Studio (Version 2024.04.2 + 764). LME models were fitted for all NVC outcome measures, including baseline and peak middle cerebral artery velocity (MCAv) and posterior cerebral artery velocity (PCAv), relative changes in MCAv and PCAv, and AUC30 conditioned on individuals. These regressions utilized P_ET_CO_2_ stages (eucapnia [reference], hypercapnia, and hypocapnia) and biological sex (female [reference] and male) as predictor variables across the cardiac cycle. Furthermore, each LME model was compared to a naive model that did not condition on individuals using a likelihood ratio (LR) test, which yielded the p-values for the LR test observed in Table 3. Afterward, a Wilcox rank-sum test was performed to compare the effect sizes of all NVC metrics (e.g., baseline, peak, relative increase, and AUC30) between male and female participants. Data are presented as mean ± 95% confidence intervals (95% CI) to estimate the population mean. Alpha was set *a priori* at 0.05.

## Results

### Physiological data

Biological sex-stratified cerebrovascular, respiratory, and cardiovascular metrics during the three “Where’s Waldo?” blocks are displayed in Table 1. All NVC outcome metrics of interest are displayed in Table 2. Table 3 shows the LME model outputs using participants as a random effect (beta coefficients and 95% CI, p-values, and LR p-values) comparing hypo- and hypercapnia to eucapnia and males to females.

**Table 1.** Biological sex-stratified cerebrovascular, respiratory, and cardiovascular metrics from 20 participants (10 females and 10 males) during a “*Where’s Waldo?*” neurovascular coupling challenge performed under three conditions (hypocapnia, eucapnia, hypercapnia).

Variable	Sex	Hypocapnia	Eucapnia	Hypercapnia
P_ET_CO_2_ (mmHg)	Female	25.9 (95% CI: 24.3, 27.6)	37.0 (95% CI: 35.3, 38.7)	52.9 (95% CI: 51.8, 53.9)
	Male	25.3 (95% CI: 23.9, 26.7)	38.0 (95% CI: 36.2, 39.9)	53.0 (95% CI: 51.4, 54.5)
Respiration Rate (BPM)	Female	17.5 (95% CI: 11.3, 23.7)	15.4 (95% CI: 11.4, 19.4)	18.8 (95% CI: 14.8, 22.8)
	Male	17.3 (95% CI: 13.2, 21.5)	16.0 (95% CI: 13.5, 18.5)	17.5 (95% CI: 15.0, 20.0)
Diastole PCAv (cm/s)	Female	18.2 (95% CI: 9.59, 26.8)	36.5 (95% CI: 25.4, 47.7)	46.5 (95% CI: 33.9, 59.1)
	Male	8.35 (95% CI: 3.39, 13.3)	26.2 (95% CI: 21.6, 30.8)	36.4 (95% CI: 31.8, 41.0)
Mean PCAv (cm/s)	Female	30.6 (95% CI: 21.6, 39.6)	47.7 (95% CI: 35.3, 60.0)	63.7 (95% CI: 47.2, 80.1)
	Male	21.6 (95% CI: 16.9, 26.3)	38.6 (95% CI: 33.3, 43.9)	51.8 (95% CI: 45.6, 58.0)
Systole PCAv (cm/s)	Female	47.4 (95% CI: 36.8, 58.1)	70.8 (95% CI: 51.7, 89.9)	87.3 (95% CI: 66.8, 107.8)
	Male	42.0 (95% CI: 34.9, 49.2)	58.9 (95% CI: 50.0, 67.7)	77.0 (95% CI: 67.3, 86.8)
Diastole MCAv (cm/s)	Female	37.9 (95% CI: 27.4, 48.5)	58.2 (95% CI: 43.8, 72.5)	85.8 (95% CI: 66.6, 105.0)
	Male	31.0 (95% CI: 27.7, 34.2)	50.2 (95% CI: 43.6, 56.7)	69.5 (95% CI: 59.2, 79.8)
Mean MCAv (cm/s)	Female	54.6 (95% CI: 40.3, 68.9)	83.5 (95% CI: 66.1, 100.9)	116.0 (95% CI: 91.6, 140.5)
	Male	46.1 (95% CI: 40.8, 51.4)	70.6 (95% CI: 60.5, 80.7)	96.0 (95% CI: 82.2, 109.7)
Systole MCAv (cm/s)	Female	83.4 (95% CI: 64.4, 102.4)	115.6 (95% CI: 92.2, 139.0)	159.3 (95% CI: 131.1, 187.6)
	Male	80.6 (95% CI: 69.1, 92.1)	108.5 (95% CI: 91.8, 125.3)	141.5 (95% CI: 122.5, 160.6)
Diastole BP (mmHg)	Female	59.5 (95% CI: 44.9, 74.2)	68.9 (95% CI: 59.0, 78.8)	73.7 (95% CI: 64.2, 83.2)
	Male	31.0 (95% CI: 27.7, 34.2)	50.2 (95% CI: 43.6, 56.7)	69.5 (95% CI: 59.2, 79.8)
MAP (mmHg)	Female	77.5 (95% CI: 64.0, 91.0)	87.7 (95% CI: 76.6, 98.8)	94.4 (95% CI: 84.0, 104.8)
	Male	76.5 (95% CI: 66.5, 86.5)	78.2 (95% CI: 66.1, 90.2)	86.2 (95% CI: 74.5, 98.0)
Systole BP (mmHg)	Female	118.8 (95% CI: 103.0, 134.7)	128.8 (95% CI: 111.2, 146.3)	140.1 (95% CI: 122.9, 157.3)
	Male	115.7 (95% CI: 105.9, 125.5)	119.5 (95% CI: 103.7, 135.4)	134.0 (95% CI: 118.0, 150.0)
Heart Rate (bpm)	Female	84.4 (95% CI: 75.1, 93.7)	79.8 (95% CI: 68.7, 90.8)	83.3 (95% CI: 72.2, 94.5)
	Male	76.4 (95% CI: 69.4, 83.4)	68.4 (95% CI: 62.3, 74.5)	72.8 (95% CI: 65.6, 79.9)

Data are displayed as mean (95% CI: confidence intervals). End-tidal carbon dioxide partial pressure (P_ET_CO_2_), breaths per minute (BPM), posterior cerebral artery velocity (PCAv), middle cerebral artery velocity (MCAv), centimeters per second (cm/s), blood pressure (BP), millimeters of mercury (mmHg), beats per minute (bpm).

**Table 2.** Absolute neurovascular coupling metrics from a *“Where’s Waldo?*” search paradigm performed under three conditions (hypocapnia, eucapnia, hypercapnia) in 20 participants (10 female and 10 male).

Vessel	Cardiac	Variable	Sex	Hypocapnia	Eucapnia	Hypercapnia
MCA	Diastole	AUC (cm/s/30s)	Female	54.2 (95% CI: 9.0, 99.3)	56.8 (95% CI: 18.5, 95.0)	−72.2 (95% CI: −113.6, −30.8)
			Male	23.3 (95% CI: −0.2, 46.8)	75.0 (95% CI: 26.4, 123.6)	−6.6 (95% CI: −49.1, 35.9)
		Baseline (cm/s)	Female	36.9 (95% CI: 26.8, 47.0)	58.8 (95% CI: 45.9, 71.7)	87.2 (95% CI: 67.8, 106.6)
			Male	29.3 (95% CI: 26.9, 31.8)	45.8 (95% CI: 39.9, 51.8)	67.0 (95% CI: 57.2, 76.9)
		Peak (cm/s)	Female	43.3 (95% CI: 30.7, 56.0)	68.0 (95% CI: 52.8, 83.2)	91.6 (95% CI: 70.4, 112.8)
			Male	33.4 (95% CI: 30.0, 36.8)	52.4 (95% CI: 46.0, 58.9)	71.6 (95% CI: 61.8, 81.4)
		Relative Increase (%)	Female	17.3 (95% CI: 10.1, 24.4)	16.1 (95% CI: 11.3, 20.8)	4.8 (95% CI: 2.7, 6.8)
			Male	13.8 (95% CI: 9.8, 17.8)	14.7 (95% CI: 9.6, 19.9)	7.3 (95% CI: 4.0, 10.5)
	Mean	AUC (cm/s/30s)	Female	76.4 (95% CI: 21.9, 131.0)	84.6 (95% CI: 40.3, 129.0)	−66.4 (95% CI: −117.1, −15.7)
			Male	32.4 (95% CI: 2.1, 62.7)	89.4 (95% CI: 38.0, 140.7)	−3.6 (95% CI: −45.6, 38.4)
		Baseline (cm/s)	Female	53.0 (95% CI: 39.5, 66.6)	81.5 (95% CI: 64.7, 98.2)	117.3 (95% CI: 93.0, 141.7)
			Male	42.9 (95% CI: 38.7, 47.1)	64.4 (95% CI: 55.0, 73.9)	91.2 (95% CI: 79.0, 103.4)
		Peak (cm/s)	Female	60.9 (95% CI: 43.1, 78.6)	92.2 (95% CI: 73.0, 111.4)	121.6 (95% CI: 95.8, 147.3)
			Male	48.1 (95% CI: 42.5, 53.6)	71.7 (95% CI: 61.7, 81.7)	95.2 (95% CI: 83.2, 107.2)
		Relative Increase (%)	Female	13.7 (95% CI: 8.7, 18.8)	13.4 (95% CI: 9.8, 16.9)	3.5 (95% CI: 1.2, 5.7)
			Male	11.8 (95% CI: 8.7, 14.8)	11.6 (95% CI: 7.5, 15.7)	4.7 (95% CI: 2.5, 6.9)
	Systole	AUC (cm/s/30s)	Female	98.6 (95% CI: 33.1, 164.0)	138.7 (95% CI: 84.3, 193.1)	−3.4 (95% CI: −45.0, 38.2)
			Male	41.0 (95% CI: 9.6, 72.4)	123.4 (95% CI: 74.6, 172.2)	21.0 (95% CI: −40.2, 82.2)
		Baseline (cm/s)	Female	81.1 (95% CI: 63.6, 98.6)	116.8 (95% CI: 96.6, 136.9)	158.9 (95% CI: 130.5, 187.2)
			Male	75.8 (95% CI: 63.9, 87.8)	100.6 (95% CI: 83.4, 117.8)	133.6 (95% CI: 116.4, 150.9)
		Peak (cm/s)	Female	92.5 (95% CI: 67.5, 117.4)	129.2 (95% CI: 105.7, 152.7)	165.2 (95% CI: 137.2, 193.2)
			Male	83.7 (95% CI: 70.4, 97.1)	110.3 (95% CI: 92.4, 128.2)	139.7 (95% CI: 121.6, 157.8)
		Relative Increase (%)	Female	12.5 (95% CI: 7.1, 17.9)	10.5 (95% CI: 8.2, 12.8)	4.3 (95% CI: 1.3, 7.2)
			Male	10.6 (95% CI: 4.3, 16.9)	9.9 (95% CI: 7.4, 12.5)	4.6 (95% CI: 1.8, 7.4)
PCA	Diastole	AUC (cm/s/30s)	Female	114.7 (95% CI: 55.2, 174.2)	165.1 (95% CI: 92.0, 238.2)	0.8 (95% CI: −44.4, 46.1)
			Male	100.9 (95% CI: 48.4, 153.4)	169.0 (95% CI: 104.2, 233.8)	48.2 (95% CI: 6.4, 90.0)
		Baseline (cm/s)	Female	16.0 (95% CI: 7.9, 24.1)	30.2 (95% CI: 21.3, 39.1)	46.0 (95% CI: 32.8, 59.1)
			Male	6.8 (95% CI: 3.0, 10.6)	22.2 (95% CI: 18.0, 26.4)	34.5 (95% CI: 29.4, 39.7)
		Peak (cm/s)	Female	24.7 (95% CI: 16.5, 32.8)	40.6 (95% CI: 28.9, 52.3)	50.1 (95% CI: 37.0, 63.2)
			Male	14.4 (95% CI: 8.9, 19.9)	31.6 (95% CI: 27.3, 35.9)	39.6 (95% CI: 33.7, 45.5)
		Relative Increase (%)	Female	109.5 (95% CI: 24.2, 194.9)	37.9 (95% CI: 22.2, 53.6)	10.2 (95% CI: 5.8, 14.6)
			Male	129.0 (95% CI: 70.1, 188.0)	46.8 (95% CI: 27.9, 65.7)	14.9 (95% CI: 9.5, 20.3)
	Mean	AUC (cm/s/30s)	Female	119.0 (95% CI: 73.1, 165.0)	188.1 (95% CI: 104.7, 271.5)	13.0 (95% CI: −38.8, 64.9)
			Male	99.9 (95% CI: 65.0, 134.8)	179.0 (95% CI: 107.1, 250.8)	52.2 (95% CI: 7.9, 96.5)
		Baseline (cm/s)	Female	28.1 (95% CI: 19.4, 36.8)	42.8 (95% CI: 32.0, 53.5)	62.7 (95% CI: 45.6, 79.8)
			Male	19.5 (95% CI: 15.1, 24.0)	33.0 (95% CI: 27.3, 38.7)	48.5 (95% CI: 41.1, 55.9)
		Peak (cm/s)	Female	35.6 (95% CI: 26.2, 45.0)	53.8 (95% CI: 39.6, 68.1)	67.0 (95% CI: 50.3, 83.7)
			Male	25.7 (95% CI: 21.0, 30.4)	42.4 (95% CI: 35.9, 48.8)	53.5 (95% CI: 45.5, 61.5)
		Relative Increase (%)	Female	33.0 (95% CI: 12.5, 53.4)	25.7 (95% CI: 21.1, 30.3)	7.8 (95% CI: 4.4, 11.1)
			Male	36.5 (95% CI: 19.6, 53.5)	30.1 (95% CI: 19.3, 40.8)	10.7 (95% CI: 6.2, 15.2)
	Systole	AUC (cm/s/30s)	Female	115.5 (95% CI: 59.1, 171.9)	236.3 (95% CI: 139.5, 333.2)	48.2 (95% CI: −27.8, 124.2)
			Male	74.7 (95% CI: 28.8, 120.6)	213.4 (95% CI: 132.9, 293.9)	66.6 (95% CI: 11.0, 122.2)
		Baseline (cm/s)	Female	45.0 (95% CI: 35.2, 54.7)	60.4 (95% CI: 47.3, 73.4)	85.1 (95% CI: 63.5, 106.7)
			Male	39.0 (95% CI: 31.6, 46.5)	51.2 (95% CI: 42.4, 60.0)	70.7 (95% CI: 60.2, 81.3)
		Peak (cm/s)	Female	53.2 (95% CI: 41.1, 65.3)	73.6 (95% CI: 56.7, 90.4)	90.9 (95% CI: 70.2, 111.6)
			Male	45.0 (95% CI: 36.5, 53.4)	62.7 (95% CI: 51.7, 73.7)	78.0 (95% CI: 65.6, 90.4)
		Relative Increase (%)	Female	18.3 (95% CI: 10.7, 25.8)	21.7 (95% CI: 17.3, 26.1)	7.8 (95% CI: 3.9, 11.8)
			Male	14.8 (95% CI: 8.5, 21.1)	22.0 (95% CI: 14.9, 29.2)	9.2 (95% CI: 5.2, 13.2)

Data are displayed as mean (95% CI: confidence intervals). Middle cerebral artery (MCA), posterior cerebral artery (PCA), AUC (Area under the curve), centimetres per second over the first 30 seconds (cm/s/30s), centimetres per second (cm/s), percentage (%).

**Table 3.** Linear mixed-effects model outputs using participants as random effects for all neurovascular coupling metrics of interest in a 20 participants (10 females and 10 males) study using the “*Where’s Waldo*” visual search paradigm and included stage (eucapnia as reference) and sex (female as reference).

Variable	Vessel	Cardiac	Hypocapnia	Hypercapnia	Sex
Baseline(cm/s)	PCA	Systole	−***13.8 (***−***20.7,*** −***6.9); p < 0.001; LR p < 0.001***	***22.1 (15.2, 29.1); p < 0.001; LR p < 0.001***	−9.8 (−24.7, 5.0); p = 0.178; LR p < 0.001
		Mean	−***14.1 (***−***19.3,*** −***8.8); p < 0.001; LR p < 0.001***	***17.7 (12.5, 23.0); p < 0.001; LR p < 0.001***	−10.8 (−22.4, 0.8); p = 0.063; LR p < 0.001
		Diastole	−***14.8 (***−***18.6,*** −***11.0); p < 0.001; LR p < 0.001***	***14.1 (10.3, 17.9); p < 0.001; LR p < 0.001***	−***9.5 (***−***19.0,*** −***0.0); p = 0.048; LR p < 0.001***
	MCA	Systole	−***30.2 (***−***38.7,*** −***21.7); p < 0.001; LR p < 0.001***	***37.6 (29.1, 46.0); p < 0.001; LR p < 0.001***	−15.6 (−39.5, 8.4); p = 0.186; LR p < 0.001
		Mean	−***25.0 (***−***32.2,*** −***17.8); p < 0.001; LR p < 0.001***	***31.3 (24.1, 38.5); p < 0.001; LR p < 0.001***	−17.8 (−35.6, 0.1); p = 0.050; LR p < 0.001
		Diastole	−***19.2 (***−***25.1,*** −***13.4); p < 0.001; LR p < 0.001***	***24.8 (18.9, 30.7); p < 0.001; LR p < 0.001***	−13.5 (−27.2, 0.1); p = 0.051; LR p < 0.001
Peak(cm/s)	PCA	Systole	−***19.1 (***−***25.7,*** −***12.4); p < 0.001; LR p < 0.001***	***16.3 (9.6, 23.0); p < 0.001; LR p < 0.001***	−10.7 (−27.7, 6.4); p = 0.202; LR p < 0.001
		Mean	−***17.5 (***−***22.4,*** −***12.5); p < 0.001; LR p < 0.001***	***12.1 (7.2, 17.1); p < 0.001; LR p < 0.001***	−11.6 (−24.7, 1.4); p = 0.075; LR p < 0.001
		Diastole	−***16.6 (***−***20.6,*** −***12.5); p < 0.001; LR p < 0.001***	***8.8 (4.8, 12.8); p < 0.001; LR p < 0.001***	−9.9 (−20.5, 0.6); p = 0.061; LR p < 0.001
	MCA	Systole	−***31.7 (***−***40.1,*** −***23.2); p < 0.001; LR p < 0.001***	***32.7 (24.2, 41.2); p < 0.001; LR p < 0.001***	−17.7 (−44.6, 9.2); p = 0.180; LR p < 0.001
		Mean	−***27.5 (***−***34.3,*** −***20.7); p < 0.001; LR p < 0.001***	***26.5 (19.7, 33.3); p < 0.001; LR p < 0.001***	−19.9 (−40.3, 0.5); p = 0.054; LR p < 0.001
		Diastole	−***21.8 (***−***27.4,*** −***16.2); p < 0.001; LR p < 0.001***	***21.4 (15.8, 27.0); p < 0.001; LR p < 0.001***	−15.2 (−30.9, 0.6); p = 0.057; LR p < 0.001
RelativeIncrease	PCA	Systole	−***5.3 (***−***9.2,*** −***1.4); p = 0.007; LR p < 0.001***	−***13.4 (***−***17.3,*** −***9.4); p < 0.001; LR p < 0.001***	−0.6 (−6.7, 5.5); p = 0.840; LR p < 0.001
(percent)		Mean	6.9 (−2.6, 16.3); p = 0.131; LR p < 0.001	−***18.7 (***−***28.1,*** −***9.2); p < 0.001; LR p < 0.001***	3.6 (−7.8, 15.0); p = 0.511; LR p < 0.001
		Diastole	**76.9 (39.5, 114.4); p < 0.001; LR p < 0.001**	−29.8 (−67.2, 7.6); p = 0.101; LR p < 0.001	11.1 (−25.9, 48.0); p = 0.534; LR p < 0.001
	MCA	Systole	1.4 (−2.3, 5.0); p = 0.444; LR p = 0.001	−***5.8 (***−***9.4,*** −***2.1); p = 0.002; LR p = 0.001***	−0.7 (−3.7, 2.3); p = 0.624; LR p = 0.001
		Mean	0.3 (−2.6, 3.2); p = 0.843; LR p < 0.001	−***8.4 (***−***11.3,*** −***5.5); p < 0.001; LR p < 0.001***	−0.8 (−4.1, 2.4); p = 0.592; LR p < 0.001
		Diastole	0.1 (−3.5, 3.8); p = 0.938; LR p < 0.001	−***9.4 (***−***13.0,*** −***5.7); p < 0.001; LR p < 0.001***	−0.8 (−5.4, 3.8); p = 0.719; LR p < 0.001
AUC(cm/s/30s)	PCA	Systole	−**129.8 (**−**187.3,** −**72.2); p < 0.001; LR p < 0.001**	−**167.5 (**−**225.0,** −**109.9); p < 0.001; LR p < 0.001**	−15.1 (−80.2, 49.9); p = 0.629; LR p < 0.001
		Mean	−**74.1 (**−**122.7,** −**25.5); p = 0.003; LR p < 0.001**	−**150.9 (**−**199.5,** −**102.3); p < 0.001; LR p < 0.001**	3.6 (−48.3, 55.5); p = 0.884; LR p < 0.001
		Diastole	−**59.2 (**−**106.7,** −**11.8); p = 0.012; LR p < 0.001**	−**142.5 (**−**190.0,** −**95.0); p < 0.001; LR p < 0.001**	12.5 (−39.6, 64.6); p = 0.618; LR p < 0.001
	MCA	Systole	−**61.3 (**−**102.0,** −**20.6); p = 0.003; LR p < 0.001**	−**122.3 (**−**163.0,** −**81.6); p < 0.001; LR p < 0.001**	−16.1 (−67.5, 35.2); p = 0.514; LR p < 0.001
		Mean	−**32.6 (**−**72.6, 7.4); p = 0.093; LR p < 0.001**	−**122.0 (**−**162.0,** −**82.0); p < 0.001; LR p < 0.001**	7.8 (−36.5, 52.1); p = 0.713; LR p < 0.001
		Diastole	−**27.1 (**−**62.5, 8.2); p = 0.113; LR p < 0.001**	−**105.3 (**−**140.6,** −**69.9); p < 0.001; LR p < 0.001**	17.6 (−21.4, 56.6); p = 0.351; LR p < 0.001

Data are displayed as mean (95% confidence intervals); p-value; LR p-value. Middle cerebral artery (MCA), posterior cerebral artery (PCA), centimeters per second (cm/s). Bold denotes significant relationship in the linear regression outputs.

**Figure 1.**
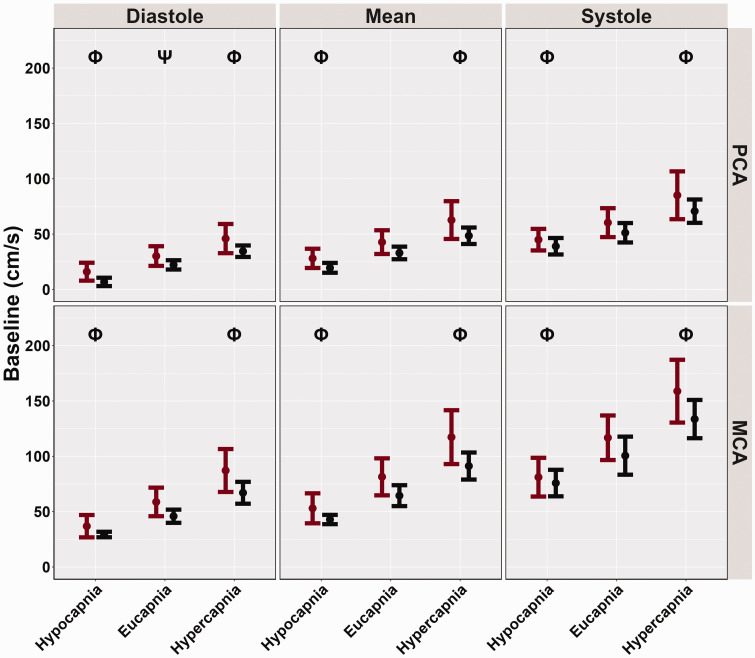
Baseline cerebral blood velocities (cm/s) obtained from participants’ posterior cerebral artery (PCA) and middle cerebral artery (MCA) across the cardiac cycle during a “Where's Waldo” neurovascular coupling (NVC) challenge. The NVC challenge was performed under three separate conditions: hypocapnia, eucapnia, and hypercapnia. The data are categorized by sex, with females represented in red (n = 10) and males in black (n = 10). The Phi symbol (Φ) indicates a stage significantly different from the eucapnia stage, determined by linear regressions with stages (eucapnia as reference) and sex (female as reference) predictor variables. The Psi (Ψ) highlights sex differences observed across the three stages (hypocapnia, eucapnia, hypercapnia).

**Figure 2.**
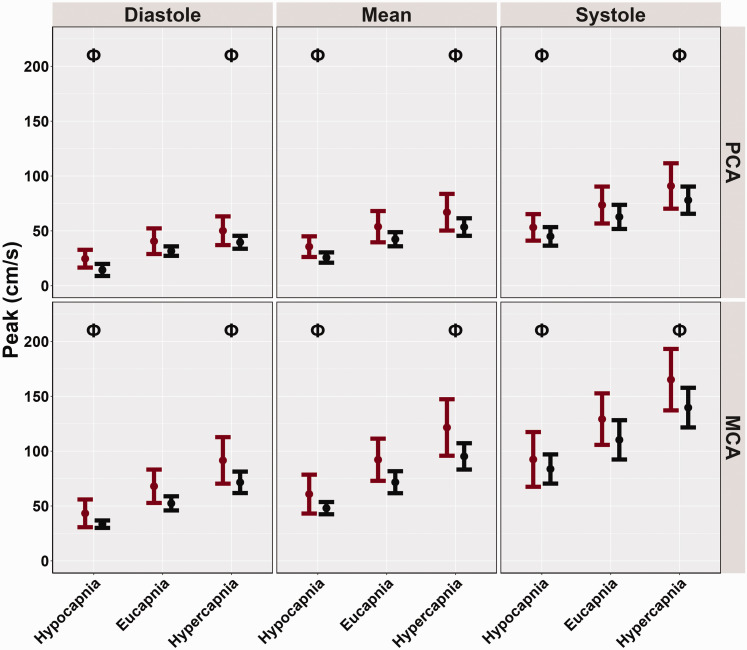
Peak cerebral blood velocities (cm/s) obtained from participants’ posterior cerebral artery (PCA) and middle cerebral artery (MCA) across the cardiac cycle during a “Where's Waldo” neurovascular coupling (NVC) challenge. The NVC challenge was performed under three separate conditions: hypocapnia, eucapnia, and hypercapnia. The data are categorized by sex, with females represented in red (n = 10) and males in black (n = 10). The Phi symbol (Φ) indicates a stage significantly different from the eucapnia stage, determined by linear regressions with stages (eucapnia as reference) and sex (female as reference) predictor variables. The Psi (Ψ) highlights sex differences observed across the three stages (hypocapnia, eucapnia, hypercapnia).

Compared to the eucapnic conditions (reference), baseline, and peak MCAv and PCAv were attenuated across the cardiac cycle during hypocapnia (all *p < 0.001,* all *LR p < 0.001*) (Table 3 and Figures 1 and 2). These metrics were all greater across the cardiac cycle during hypercapnia (all *p < 0.001,* all *LR p < 0.001*) (Table 3 and Figures 1 and 2). However, biological sex differences were only observed across conditions for PCA diastole metrics (*p = 0.048, LR p < 0.001*) where females displayed greater PCA diastole measures compared to males (Table 3, Figures 1 and 2). Conversely, all other peak and baseline metrics did not display significant differences, (all *p > 0.050*) (Table 3, Figures 1 and 2). Across conditions and the cardiac cycle, baseline measures indicated all biological sex effect sizes were below 0.263. Similarly, for peak metrics, across conditions and the cardiac cycle, all biological sex effect sizes were below 0.273.

**Figure 3.**
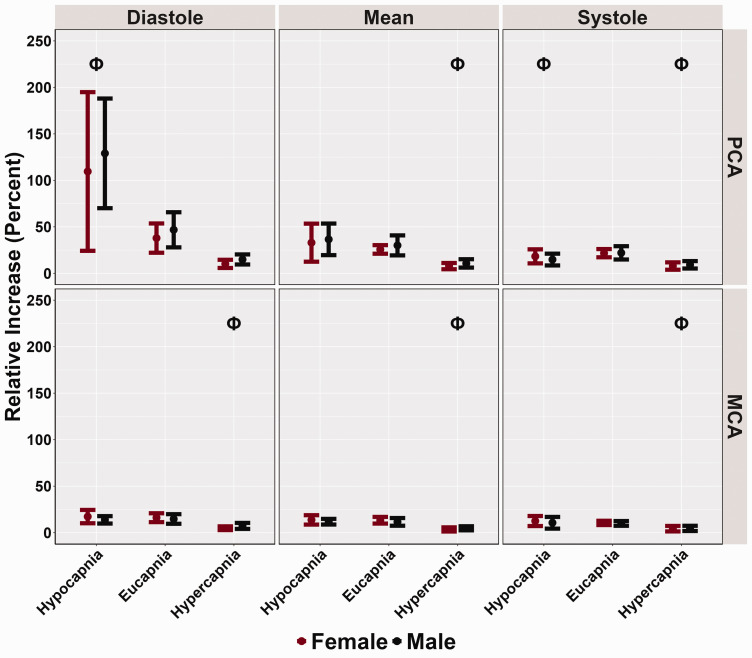
Relative increase (%) obtained from participants’ posterior cerebral artery (PCA) and middle cerebral artery (MCA) across the cardiac cycle during a “Where's Waldo” neurovascular coupling (NVC) challenge. The NVC challenge was performed under three separate conditions: hypocapnia, eucapnia, and hypercapnia. The data are categorized by sex, with females represented in red (n = 10) and males in black (n = 10). The Phi symbol (Φ) indicates a stage significantly different from the eucapnia stage, determined by linear regressions with stages (eucapnia as reference) and sex (female as reference) predictor variables. The Psi (Ψ) highlights sex differences observed across the three stages (hypocapnia, eucapnia, hypercapnia).

The relative increase in PCAv was greater during hypocapnia diastole (*p < 0.001*), lower in systole (*p = 0.007*), and similar in mean (*p = 0.131*) compared to eucapnic conditions (Table 3 and Figure 3). No differences in MCA relative increase were noted across the cardiac cycle when comparing hypocapnic to eucapnic trials (all *p > 0.444*) (Table 3 and Figure 3). During hypercapnic stages, the relative PCAv increase was lower during systole *(p < 0.001, LR p < 0.001*) and mean *(p < 0.001, LR p < 0.001*) (Table 3 and Figure 3). However, no differences in hypercapnic diastole PCAv were found compared to eucapnic relative increase (*p = 0.101*) (Table 3 and Figure 3). Throughout the hypercapnic trials, the relative MCAv increase was lower across the cardiac cycle (all *p < 0.002,* all *LR p < 0.001*) (Table 3 and Figure 3). No biological sex differences were recorded across conditions and the cardiac cycle in both PCAv (all *p > 0.511*) and MCAv (all *p > 0.592*) relative increase metrics (Table 3 and Figure 3). Across conditions and across the cardiac cycle, relative increase metrics displayed small biological sex effect sizes, as all effect sizes were discovered to be below 0.107.

**Figure 4.**
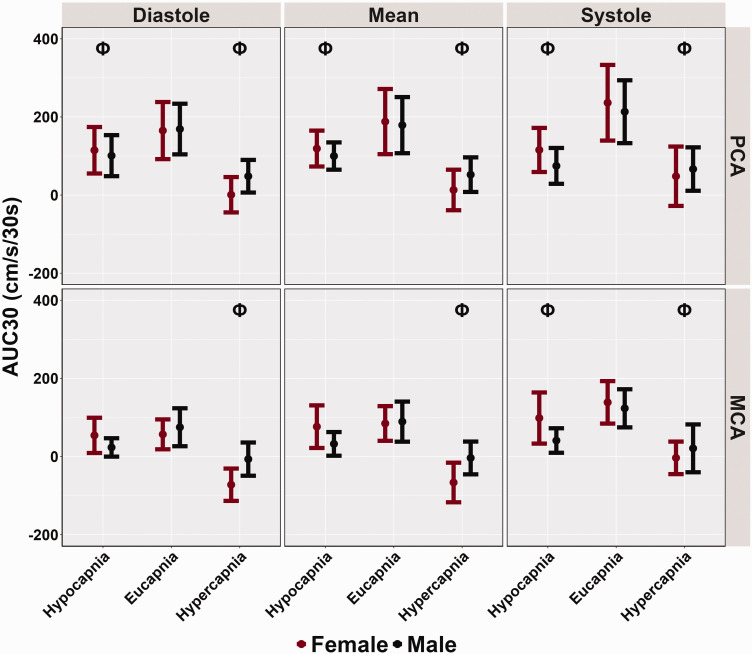
Area under the curve during 30 seconds of task engagement (AUC30) obtained from participants’ posterior cerebral artery (PCA) and middle cerebral artery (MCA) across the cardiac cycle during a “Where's Waldo” neurovascular coupling (NVC) challenge. The NVC challenge was performed under three separate conditions: hypocapnia, eucapnia, and hypercapnia. The data are categorized by sex, with females represented in red (n = 10) and males in black (n = 10). The Phi symbol (Φ) indicates a stage significantly different from the eucapnia stage, determined by linear regressions with stages (eucapnia as reference) and sex (female as reference) predictor variables. The Psi (Ψ) highlights sex differences observed across the three stages (hypocapnia, eucapnia, hypercapnia).

The PCAv AUC30 was reduced across the cardiac cycle during both hypocapnia (all *p < 0.012*, all *LR p < 0.*001) and hypercapnia (all *p < 0.001,* all *LR p < 0.001*) compared to eucapnic trials (Table 3 and Figure 4). Compared to the reference stage, the systolic MCAv AUC30 was lower during hypocapnic conditions (*p = 0.003, LR p < 0.001*) and remained similar for diastolic (*p = 0.113*) and mean (*p = 0.093*) components of the cardiac cycle (Table 3 and Figure 4). During hypercapnic trials, MCAv AUC30 was lower across the cardiac cycle when compared to eucapnic trials (all *p < 0.001,* all *LR p < 0.001*) (Table 3 and Figure 4). No biological sex differences were found within AUC30 metrics across the cardiac cycle for PCAv (all *p > 0.618*) and MCAv (all *p > 0.351*) (Table 3 and Figure 4). Finally, across all conditions and the cardiac cycle, small biological sex effect sizes were noted, as all effect sizes were determined to be below 0.115.

## Discussion

The primary findings from this investigation were three-fold: 1) During hypocapnic trials, both MCAv and PCAv AUC30 were reduced, 2) Throughout the hypercapnic stage, PCAv AUC30 was lower across the cardiac cycle, and in the systolic component of the MCAv AUC30, with no changes reported in mean and diastole components, and 3) When accounting for individual variability through the LME modeling, minimal sex differences were observed in any NVC metrics (besides greater baseline diastolic PCA) across all conditions and across the cardiac cycle (Table 3).

### Comparisons with previous literature

Numerous studies have explored temporal NVC responses under eucapnic conditions.^5,17,18,56–59^ Previous research has shown females have higher levels of resting CBF.^60–63^ In the current investigation, females displayed greater CBv for all baseline and peak metrics across the cardiac cycle; however, only PCA diastolic baseline measures showed significant biological sex differences (Table 3, Figures 1 and 2). Additionally, no disparities were found in the relative percent increase and AUC30 metrics, which characterize the NVC response (Table 3, Figures 3 and 4). These findings somewhat contrast with previous literature,^14,28,58^ which reports biological sex differences in baseline and peak CBv but no differences in relative increase or AUC30, despite using the same complex visual scene search paradigm. Furthermore, recent research has demonstrated that sex differences may depend on age as these factors jointly model the NVC response.^59^ The difference in findings is likely due to the statistical analysis performed in each study. The current investigation employed LME modeling conditioning on individual, which better controls for within subject differences which may confound investigations of sex differences when within-individual variability is not considered. Furthermore, several male participants displayed higher than expected CBv, which may explain the lack of observed biological sex differences. CBv is known to be elevated in females compared to males,^64,65^ thus the unexpected CBv in the male participants may have disguised the effect of sex in this sample. A previous study conducted by Alwatban and colleagues^66^ indicates that for 18–30 year olds, the average MCAv for females is 70.5 +/− 12.7, whereas for males it is 59.5 +/− 10.3. The current investigation used a similar age population, however, five males displayed larger than expected CBv, with an average mean MCAv of 81.89 cm/s, and an average mean PCAv of 41.96 cm/s. Finally, as the study did not have sufficient power to investigate biological sex differences across the individual stages, the three conditions were combined to examine these discrepancies. This may have influenced the results and could partially explain the differences observed in baseline and peak metrics in the current results (Table 3) compared with compared to previous literature.^14,28^ Nevertheless, the LME outputs suggests that while baseline and peak, diastolic and mean MCAv and PCAv did not reach the conventional significance of 0.05 with *p-values* ranging from 0.048 and 0.075 (Table 3). However, the consistent trend across baseline and peak diastolic measures points toward a probable sex difference (Table 3), consistent with previous work. In contrast, the relative increase in CBv and AUC *p-values* (ranging from 0.351 to 0.719) are consistently far from this threshold, indicating a lack of evidence for sex differences in those comparisons (Table 3).

Previous literature investigating the hypocapnic NVC response in both sexes observed reduced peak responses during hypocapnia compared to eucapnia, though with differing levels of hypocapnia (Δ −5–10 mmHg via hyperventilation compared to PETCO clamped at 25 mmHg via end tidal forcing) and employing different visual stimuli (strobe light vs. Where’s Waldo) compared to the present study.^27,28^ While this research included both sexes, the data was aggregated and sex differences were not explored^27^ These studies, which both employed a visual stimulus, differed in that one found no change in mean PCAv and area under the curve (AUC) between conditions^27^ while the other showed a reduced response.^28^ However, this study differed from Bader and colleagues in the AUC30 findings, as PCAv AUC30 was observed to be lower across the cardiac cycle (Table 3 and Figure 4). These differences could be attributed to the P_ET_CO_2_ metrics utilized, as the current investigation clamped P_ET_CO_2_ at 25 mmHg for the hypocapnic trials (−15 Torr), while the aforementioned study used −10 Torr for the largest hypocapnic stimulus. Moreover, the two investigations employed different NVC challenges, with Bader et al.^27^ using a visual stimulus with strobe lights, whereas the current investigation utilized a complex visual scene-search paradigm (*“Where’s Waldo?”*). A more complex and demanding task is likely to recruit a larger neurological response, in turn increasing the neurometabolic demand which elicits a larger compensation by the intracranial arteries via the neurovascular coupling response, thus enhancing the signal-to-noise ratio.^16,28^ While the results from Szabo and colleagues^26^ are consistent with the findings of the current investigation (Table 3, Figures 1 and 2), and those from Bader and colleagues,^27^ the relative increase was reported to be reduced during hypocapnic conditions compared to eucapnic trials.^26^ These findings diverge slightly from the present investigation, which showed that during hypocapnia, the PCAv relative increase was greater in diastole, similar in mean, and reduced in systole, while no differences were found within MCAv relative increase (Table 3 and Figure 3). The differences between findings for the mean relative increase may be attributed to the different NVC challenges used, as both studies employed similar P_ET_CO_2_ levels for hypocapnic and eucapnic conditions.^26^

Additionally, although there is limited research on the effects of hypercapnia on NVC using visual stimuli, a study conducted by Davies and colleagues^67^ employed a patterned counting task as well as a cognitive backwards counting stimulus to study NVC during hypercapnia. They induced hypercapnia using a 5% CO_2_ gas inhalation and measured % change in the NVC response do the gas inhalation.^67^ The Davies study observed a much smaller change during hypercapnia (3–15% depending on task) than the present study, likely due to the methodological differences in the hypercapnic stimuli and the NVC task.^67^ The present results may be more striking due to the tighter control of PETCO_2_ using end tidal forcing, and the more robust NVC response to the ‘Where’s Waldo’ visual task. In another study, Maggio and colleagues^24^ employed a passive motor paradigm involving repetitive flexion and extension of participants’ elbows under eucapnic and hypercapnic conditions. Similarly, biological sex differences were not reported, as data was collapsed across biological sexes. In their protocol, hypercapnia was induced by breathing a mixture of 5% CO_2_ through a mask, and bilateral MCAs were monitored to observe changes in CBv which found that AUC30 was lower during the hypercapnic NVC challenge.^24^ These results align with the findings from the current investigation, which also reported lower AUC30 during hypercapnic trials in both the MCAv and PCAv and across all components of the cardiac cycle (Table 3 and Figure 4). More recently, a study examined the influence of age on hypercapnic and hypocapnic NVC during a cognitive paradigm.^67^ In agreement with the current findings, Davies and colleagues observed a reduction in the NVC response during hypercapnia (elicited by 5% CO_2_ gas inhalation) and hypocapnia (hyperventilation). Additionally, they reported no absolute change differences, but found that middle aged participants displayed a ‘younger’ NVC response during hypercapnia and an ‘older’ response during hypocapnia.^67^

### Physiological underpinnings of the tasks

While the NVC response is mostly nitric oxide-driven, recent pharmacological blockade studies have demonstrated that unknown mechanisms account for roughly 1/3 of the response.^68^, animal research has suggested that CO_2_, a metabolite on neural metabolism, mediates the neurovascular coupling response, given that local CO_2_ saturation abolishes the neurovascular coupling response.^69^ In the present study, hypercapnia induced global increases in both baseline and peak (Table 3, Figures 1 and 2) CBv. However, there was a decrease in relative percent increase and AUC30 during hypercapnic trials, showing a smaller NVC response (Table 3, Figures 3 and 4). The increase in baseline and peak CBv is attributed to hypercapnic vasodilation, which occurs due to increased inspiration of CO_2_, H^+^ ions, reductions in pH, and relaxation of smooth muscle cells.^70^ One possible physiological reason for the decreased relative percent increase and AUC30 noted during the *“Where’s Waldo?*” NVC task under hypercapnic conditions is that hypercapnia induces considerable vasodilation, restricting cerebral blood vessels from further dilating in response to localized activation changes.^71,72^ This physiological phenomenon is referred to as “*vasodilatory reserve*” and indicates that the NVC response and cerebrovascular reactivity response overlap in mechanism, perhaps related to the observation that the majority of glucose consumed by neurons during a period of neuronal excitation becomes CO_2_.^69,73^ However, the relative increase and AUC30 were still elevated compared to the baseline during the hypercapnic trials (Table 3, Figures 3 and 4), suggesting that, in accordance with the animal literature, other mechanisms are also in part influencing the NVC response. This demonstrates the complex interplay of various physiological factors in regulating CBv during altered P_ET_CO_2_ levels.

Hypocapnia, however, may increase the vasodilatory reserve. In the present study, during hypocapnic trials, both baseline and peak CBv were decreased (Table 3, Figures 1 and 2). Additionally, the hypocapnic condition induced greater relative percent increases in PCA diastolic CBv, reduced systolic relative percent increases, and reductions in AUC30 compared to eucapnic trials (Table 3, Figures 3 and 4). Previous studies have demonstrated that hypocapnia induces reductions in CBF due to its vasoconstrictive effects on the cerebral vasculature.^74^ This vasoconstrictive effect of hypocapnia may inhibit the NVC response, contributing to the reductions in AUC30 observed during NVC challenges (Table 3 and Figure 4). Additionally, decreases in cerebral perfusion and oxygen delivery induced by hypocapnia may result in brain tissue hypoxia, further diminishing the NVC response.^75^

Recent evidence suggests that blood pressure changes may influence the NVC response in humans, suggesting an interaction between dynamic Cerebral Autoregulation (dCA) and NVC. A study by Ladthavorlaphatt et al.^76^ characterized individuals into “responders” or “non-responders” based on their dCA response to neural activation. This stratification demonstrated that some individual’s vascular responses may be less efficient in regulating blood flow during moments of heightened neural demand, especially under variable CO_2_ levels. However, an important distinction between studies is that Ladthavorlaphatt et al.^76^ obtained their estimates from a singular trial, across 3-minutes (1-minute rest period, 1-minute task activation, 1-minute recovery period). Observations of a singular trial would indeed be more influenced by changes in blood pressure and other physiological contaminations. Moreover, these authors used a cognitive task to elicit the NVC response,^45^ which does not elicit as robust a response as the visual paradigm employed in the current investigation. Therefore, while it is the case that dCA metrics such as autoregulation index vary by sex and end-tidal CO_2_ level,^77^ in the present study, the combination of repetitive trials and a more engaging task would attenuate the confounding ‘noise’ of other physiological fluctuations such as the dCA response to changing blood pressure. Nonetheless, had the analysis been conducted over time using a moving average or similar techniques, accounting for blood pressure fluctuations would have been essential to accurately interpret the NVC response. This approach would allow for a clearer understanding of how dCA interacts with NVC, particularly under conditions of dynamic blood pressure changes, which warrants further investigation.

Furthermore, while the demands of the hypercapnic condition in the current study may have elicited an increase in stress, associated with a relatively heightened sympathetic state, autonomic control of the cerebrovasculature is of significantly smaller magnitude compared to the NVC and CO_2_ tensions, suggesting that these effects would have negligible impacts on the observed responses.^78^ The comparison of these regulatory mechanisms is described in more detail in Figure 1 of Smith and Ainsley (2017).

### Limitations

A primary limitation of the experimental setup is the utilization of TCD to assess participants’ CBF throughout the protocol. TCD employs CBv as a proxy for CBF due to its inability to directly measure vessel diameter.^20^ Consequently, it operates under the assumption that vessel diameter remains constant throughout the protocol.^20^ Nonetheless, previous studies employing functional magnetic resonance imaging have suggested that CBv remains relatively stable within 8 mmHg of eucapnic values.^79,80^ Despite attempts to regulate P_ET_CO_2_ levels at precise targets to surpass the suggested ∼8 mmHg range (i.e., 40 mmHg for eucapnia, 55 mmHg for hypercapnia, and 25 mmHg for hypocapnia), the observed results align with prior studies, bolstering confidence in the current findings.

Furthermore, the study recruited a convenience sample from around the university including graduate students and staff. The sample of healthy, young, and likely higher socioeconomic status adults limits the generalizability of the findings to other populations such as those with brain injuries, older individuals, those with a lower socioeconomic status or people with less compliant brain blood vessels. Furthermore, since culture and ethnicity were not assessed in this study, generalisability across cultures or ethnicities cannot be inferred. Future research targeting the associations between culture, ethnicity and cerebral hemodynamics is warranted. Additionally, all participants in this study identified as cis-gendered, restricting the study’s ability to comment on the associations between gender and NVC response. Future studies should aim to recruit non-cis-gender individuals to better understand the interplay between the societal influence of gender and cerebrovascular research. Moreover, the influence of the menstrual cycle phase, hormonal contraceptive use and cardiac fitness status was not accounted for in the present study. Therefore, future studies are warranted to investigate whether the menstrual cycle phase, contraceptive use, and cardiac fitness influence NVC responses. Sex differences were collapsed across conditions, as the current study did not have the subsequent power to examine biological sex differences across all individual stages albeit the trends were consistent with previous findings.^28^ Nonetheless, each participant underwent all three conditions during a single visit, effectively serving as their own controls in eucapnic conditions across hypercapnic and hypocapnic trials. This approach minimizes the influence of potential confounding factors in task comparisons.^50^

## Conclusions

The aim of this study was to investigate the influence of CVR on NVC responses during a complex visual search task (*“Where’s Waldo?*”). The findings revealed decreases in PCAv and MCAv AUC30 throughout the cardiac cycle in both hypercapnic and hypocapnic conditions. Additionally, sex differences were only observed in baseline PCA diastole measures, with females showing higher absolute CBv. No significant differences between sexes were found in the main NVC metrics, such as relative percent increase and AUC30, across all trials. These results suggest that while both hypo- and hypercapnia have notable effects on the overall NVC response, there is minimal impact related to biological sex on these robust stimuli. The cerebrovasculature in both females and males appears to respond robustly to neural activation even under significant alterations in CO_2_ tensions. Further research is needed to differentiate between metabolic and hemodynamic changes associated with altered carbon dioxide levels and their impact on temporal NVC responses.

## Data Availability

The data supporting the findings from this project are available upon reasonable request to the corresponding author (M.G.N).

## References

[1] Rink C Khanna S. Significance of brain tissue oxygenation and the arachidonic acid cascade in stroke. Antioxid Redox Signal 2011; 14: 1889–1903. 20673202 10.1089/ars.2010.3474 PMC3078506

[2] Watts ME Pocock R Claudianos C. Brain energy and oxygen metabolism: emerging role in normal function and disease. Front Mol Neurosci 2018; 11: 216. 29988368 10.3389/fnmol.2018.00216 PMC6023993

[3] Williams LR Leggett RW. Reference values for resting blood flow to organs of man. Clin Phys Physiol Meas Off Meas 1989; 10: 187–217. 10.1088/0143-0815/10/3/001 2697487

[4] Nippert AR Biesecker KR Newman EA. Mechanisms mediating functional hyperemia in the brain. Neuroscientist 2018; 24: 73–83. 28403673 10.1177/1073858417703033 PMC5757525

[5] Phillips AA Chan FH Zheng MMZ , et al. Neurovascular coupling in humans: physiology, methodological advances and clinical implications. J Cereb Blood Flow Metab 2016; 36: 647–664. 26661243 10.1177/0271678X15617954 PMC4821024

[6] Nicolakakis N Hamel E. Neurovascular function in Alzheimer’s disease patients and experimental models. J Cereb Blood Flow Metab Off J Tab 2011; 31: 1354–1370. 10.1038/jcbfm.2011.43 PMC3130325 21468088

[7] Phillips AA Warburton DER Ainslie PN , et al. Regional neurovascular coupling and cognitive performance in those with low blood pressure secondary to high-level spinal cord injury: improved by alpha-1 agonist midodrine hydrochloride. J Cereb Blood Flow Metab Off J Tab 2014; 34: 794–801. 10.1038/jcbfm.2014.3 PMC4013775 24473484

[8] Hinzman JM Andaluz N Shutter LA , et al. Inverse neurovascular coupling to cortical spreading depolarizations in severe brain trauma. Brain J Brain 2014; 137: 2960–2972. 10.1093/brain/awu241 25154387

[9] Wright AD Smirl JD Bryk K , et al. A prospective transcranial doppler ultrasound-based evaluation of the acute and cumulative effects of sport-related concussion on neurovascular coupling response dynamics. J Neurotrauma 2017; 34: 3097–3106. 28627298 10.1089/neu.2017.5020

[10] Beishon LC Williams CAL Panerai RB , et al. The assessment of neurovascular coupling with the Addenbrooke’s cognitive examination: a functional transcranial doppler ultrasonographic study. J Neurophysiol 2018; 119: 1084–1094. 29187557 10.1152/jn.00698.2017

[11] Ladthavorlaphatt K Surti FBS Beishon LC , et al. Challenging neurovascular coupling through complex and variable duration cognitive paradigms: a subcomponent analysis. Med Eng Phys 2022; 110: 103921. 36564144 10.1016/j.medengphy.2022.103921

[12] Salinet ASM Robinson TG Panerai RB. Active, passive, and motor imagery paradigms: component analysis to assess neurovascular coupling. J Appl Physiol (1985) 2013; 114: 1406–1412. 23449939 10.1152/japplphysiol.01448.2012

[13] Salinet ASM Panerai RB Robinson TG. The longitudinal evolution of cerebral blood flow regulation after acute ischaemic stroke. Cerebrovasc Dis Extra 2014; 4: 186–197. 25298773 10.1159/000366017 PMC4176407

[14] Burma JS Wassmuth RM Kennedy CM , et al. Does task complexity impact the neurovascular coupling response similarly between males and females? Physiol Rep 2021; 9: e15020. 34514743 10.14814/phy2.15020 PMC8436054

[15] Rosengarten B Kaps M. A simultaneous EEG and transcranial doppler technique to investigate the neurovascular coupling in the human visual cortex. Cerebrovasc Dis Basel Dis 2010; 29: 211–216. 10.1159/000267840 20029192

[16] Smirl JD Wright AD Bryk K , et al. Where’s Waldo ? The utility of a complicated visual search paradigm for transcranial doppler-based assessments of neurovascular coupling. J Neurosci Methods 2016; 270: 92–101. 27291357 10.1016/j.jneumeth.2016.06.007

[17] Gommer ED Bogaarts G Martens EGHJ , et al. Visually evoked blood flow responses and interaction with dynamic cerebral autoregulation: correction for blood pressure variation. Med Eng Phys 2014; 36: 613–619. 24507691 10.1016/j.medengphy.2014.01.006

[18] Willie CK Cowan EC Ainslie PN , et al. Neurovascular coupling and distribution of cerebral blood flow during exercise. J Neurosci Methods 2011; 198: 270–273. 21459113 10.1016/j.jneumeth.2011.03.017

[19] Fierstra J Sobczyk O Battisti-Charbonney A , et al. Measuring cerebrovascular reactivity: what stimulus to use? J Physiol 2013; 591: 5809–5821. 24081155 10.1113/jphysiol.2013.259150 PMC3872753

[20] Ainslie PN Hoiland RL. Transcranial doppler ultrasound: valid, invalid, or both? J Appl Physiol (1985) 2014; 117: 1081–1083. 25257879 10.1152/japplphysiol.00854.2014

[21] Kety SS Schmidt CF. The effects of altered arterial tensions of carbon dioxide and oxygen on cerebral blood flow and cerebral oxygen consumption of normal young men. J Clin Invest 1948; 27: 484–492. 16695569 10.1172/JCI101995 PMC439519

[22] O'Connor SM Wong JD Donelan JM. A generalized method for controlling end-tidal respiratory gases during nonsteady physiological conditions. J Appl Physiol 2016; 121: 1247–1262. 10.1152/japplphysiol.00274.2016 27633735

[23] Tymko MM Ainslie PN MacLeod DB , et al. End tidal-to-arterial CO_2_ and O_2_ gas gradients at low- and high-altitude during dynamic end-tidal forcing. Am J Physiol Regul Integr Comp Physiol 2015; 308: R895–906. 25810386 10.1152/ajpregu.00425.2014

[24] Maggio P Salinet ASM Panerai RB , et al. Does hypercapnia-induced impairment of cerebral autoregulation affect neurovascular coupling? A functional TCD study. J Appl Physiol (1985) 2013; 115: 491–497. 23743398 10.1152/japplphysiol.00327.2013 PMC3742941

[25] Rosengarten B Spiller A Aldinger C , et al. Control system analysis of visually evoked blood flow regulation in humans under normocapnia and hypercapnia. Eur J Ultrasound Off Ultrasound 2003; 16: 169–175. 10.1016/s0929-8266(02)00070-8 12573785

[26] Szabo K Lako E Juhasz T , et al. Hypocapnia induced vasoconstriction significantly inhibits the neurovascular coupling in humans. J Neurol Sci 2011; 309: 58–62. 21831399 10.1016/j.jns.2011.07.026

[27] Bader TJ Leacy JK Keough JRG , et al. The effects of acute incremental hypocapnia on the magnitude of neurovascular coupling in healthy participants. Physiol Rep 2021; 9: e14952. 34350726 10.14814/phy2.14952 PMC8339533

[28] Burma JS Rattana S Johnson NE , et al. Do mean values tell the full story? Cardiac cycle and biological sex comparisons in temporally derived neurovascular coupling metrics. J Appl Physiol (1985) 2023; 134: 426–443. 36603050 10.1152/japplphysiol.00170.2022

[29] Tallon CM Barker AR Nowak-Flück D , et al. The influence of age and sex on cerebrovascular reactivity and ventilatory response to hypercapnia in children and adults. Exp Physiol 2020; 105: 1090–1101. 32333697 10.1113/EP088293

[30] World Medical Association. World medical association declaration of Helsinki: ethical principles for medical research involving human subjects. JAMA 2013; 310: 2191–2194. 24141714 10.1001/jama.2013.281053

[31] Burma JS Lapointe AP Wilson M , et al. Adolescent sport-related concussion and the associated neurophysiological changes: a systematic review. Pediatr Neurol 2024; 150: 97–106. 38006666 10.1016/j.pediatrneurol.2023.10.020

[32] Mauvais-Jarvis F Bairey Merz N Barnes PJ , et al. Sex and gender: modifiers of health, disease, and medicine. Lancet Lond Lancet 2020; 396: 565–582. 10.1016/S0140-6736(20)31561-0 PMC7440877 32828189

[33] Lapointe AP Ritchie JN Vitali RV , et al. Internal consistency of sway measures via embedded head-mounted accelerometers: implications for neuromotor investigations. Sensors (Basel) 2021; 13: 4492. 10.3390/s21134492 PMC8271381 34209391

[34] Ainslie PN Barach A Murrell C , et al. Alterations in cerebral autoregulation and cerebral blood flow velocity during acute hypoxia: rest and exercise. Am J Physiol Heart Circ Physiol 2007; 292: H976–83. 17012355 10.1152/ajpheart.00639.2006

[35] Ainslie PN Cotter JD George KP , et al. Elevation in cerebral blood flow velocity with aerobic fitness throughout healthy human ageing. J Physiol 2008; 586: 4005–4010. 18635643 10.1113/jphysiol.2008.158279 PMC2538930

[36] Smirl JD Haykowsky MJ Nelson MD , et al. Relationship between cerebral blood flow and blood pressure in long-term heart transplant recipients. Hypertension 2014; 64: 1314–1320. 25287403 10.1161/HYPERTENSIONAHA.114.04236

[37] Smirl JD Hoffman K Tzeng YC , et al. Methodological comparison of active- and passive-driven oscillations in blood pressure; implications for the assessment of cerebral pressure-flow relationships. J Appl Physiol (1985) 2015; 119: 487–501. 26183476 10.1152/japplphysiol.00264.2015 PMC4556839

[38] Burma JS Copeland P Macaulay A , et al. Dynamic cerebral autoregulation across the cardiac cycle during 8 hr of recovery from acute exercise. Physiol Rep 2020; 8: e14367. 32163235 10.14814/phy2.14367 PMC7066871

[39] Claassen JA Meel-van Den Abeelen AS Simpson DM , et al. Transfer function analysis of dynamic cerebral autoregulation: a white paper from the international cerebral autoregulation research network. J Cereb Blood Flow Metab 2016; 36: 665–680. 26782760 10.1177/0271678X15626425 PMC4821028

[40] Hamner JW Tan CO Lee K , et al. Sympathetic control of the cerebral vasculature in humans. Stroke 2010; 41: 102–109. 20007920 10.1161/STROKEAHA.109.557132 PMC2814242

[41] Hamner JW Tan CO. Relative contributions of sympathetic, cholinergic, and myogenic mechanisms to cerebral autoregulation. Stroke 2014; 45: 1771–1777. 24723314 10.1161/STROKEAHA.114.005293 PMC4102642

[42] Newel KT Burma JS Carere J , et al. Does oscillation size matter? Impact of added resistance on the cerebral pressure‐flow relationship in females and males. Physiol Rep 2022; 10: e15278. 35581899 10.14814/phy2.15278 PMC9114660

[43] Panerai RB Brassard P Burma JS , et al. Transfer function analysis of dynamic cerebral autoregulation: a CARNet white paper 2022 update. J Cereb Blood Flow Metab 2023; 43: 3–25. 35962478 10.1177/0271678X221119760 PMC9875346

[44] Zhang R Zuckerman JH Giller CA , et al. Transfer function analysis of dynamic cerebral autoregulation in humans. Am J Physiol 1998; 274: H233–41. 9458872 10.1152/ajpheart.1998.274.1.h233

[45] Ball JD Hills E Altaf A , et al. Neurovascular coupling methods in healthy individuals using transcranial doppler ultrasonography: a systematic review and consensus agreement. J Cereb Blood Flow Metab Off J Tab 2024; 44: 1409–1429. 10.1177/0271678X241270452 PMC11572172 39113406

[46] Burma JS Van Roessel RK Oni IK , et al. Neurovascular coupling on trial: how the number of trials completed impacts the accuracy and precision of temporally derived neurovascular coupling estimates. J Cereb Blood Flow Metab Off J Tab 2022; 42: 1478–1492. 10.1177/0271678X221084400 PMC9274868 35209741

[47] Handford M. Where’s Waldo?. Boston: Little, Brown and Company, 1987.

[48] Skow RJ MacKay CM Tymko MM , et al. Differential cerebrovascular CO_2_ reactivity in anterior and posterior cerebral circulations. Respir Physiol Neurobiol 2013; 189: 76–86. 23774143 10.1016/j.resp.2013.05.036

[49] Burma JS Macaulay A Copeland P , et al. Comparison of cerebrovascular reactivity recovery following high‐intensity interval training and moderate‐intensity continuous training. Physiol Rep 2020; 8: e14467. 32506845 10.14814/phy2.14467 PMC7276190

[50] Charness G Gneezy U Kuhn MA. Experimental methods: between-subject and within-subject design. J Econ Behav Organ 2012; 81: 1–8.

[51] Skow RJ Brothers RM Claassen JAHR , et al. On the use and misuse of cerebral hemodynamics terminology using transcranial doppler ultrasound: a call for standardization. Am J Physiol Heart Circ Physiol 2022; 323: H350–H357. 35839156 10.1152/ajpheart.00107.2022

[52] Willie CK Colino FL Bailey DM , et al. Utility of transcranial doppler ultrasound for the integrative assessment of cerebrovascular function. J Neurosci Methods 2011; 196: 221–237. 21276818 10.1016/j.jneumeth.2011.01.011

[53] Bagiella E Sloan RP Heitjan DF. Mixed-effects models in psychophysiology. Psychophysiology 2000; 37: 13–20. 10705763

[54] Matuschek H Kliegl R Vasishth S , et al. Balancing type I error and power in linear mixed models. J Mem Lang 2017; 94: 305–315.

[55] Thiese MS. Observational and interventional study design types; an overview. Biochem Med (Zagreb) 2014; 24: 199–210. 24969913 10.11613/BM.2014.022 PMC4083571

[56] Aaslid R. Visually evoked dynamic blood flow response of the human cerebral circulation. Stroke 1987; 18: 771–775. 3299883 10.1161/01.str.18.4.771

[57] Girouard H Iadecola C. Neurovascular coupling in the normal brain and in hypertension, stroke, and Alzheimer disease. J Appl Physiol (1985) 2006; 100: 328–335. 16357086 10.1152/japplphysiol.00966.2005

[58] Leacy JK Johnson EM Lavoie LR , et al. Variation within the visually evoked neurovascular coupling response of the posterior cerebral artery is not influenced by age or sex. J Appl Physiol (1985) 2022; 133: 335–348. 35771218 10.1152/japplphysiol.00292.2021 PMC9359642

[59] Koep JL Bond B Barker AR , et al. Sex modifies the relationship between age and neurovascular coupling in healthy adults. J Cereb Blood Flow Metab Off J Tab 2023; 43: 1254–1266. 10.1177/0271678X231167753 PMC10369153 37017422

[60] Bertsch K Hagemann D Hermes M , et al. Resting cerebral blood flow, attention, and aging. Brain Res 2009; 1267: 77–88. 19272361 10.1016/j.brainres.2009.02.053

[61] Devous MD Stokely EM Chehabi HH , et al. Normal distribution of regional cerebral blood flow measured by dynamic single-photon emission tomography. J Cereb Blood Flow Metab 1986; 6: 95–104. 3484747 10.1038/jcbfm.1986.12

[62] Lu H Xu F Rodrigue KM , et al. Alterations in cerebral metabolic rate and blood supply across the adult lifespan. Cereb Cortex N Y N 1991 2011; 21: 1426–1434. 10.1093/cercor/bhq224 PMC3097991 21051551

[63] Rodriguez G Warkentin S Risberg J , et al. Sex differences in regional cerebral blood flow. J Cereb Blood Flow Metab 1988; 8: 783–789. 3192645 10.1038/jcbfm.1988.133

[64] Johnson NE Burma JS Seok J , et al. Influence of sex on the reliability of cerebral blood velocity regulation during lower body negative pressure and supine cycling with considerations of the menstrual cycle. Physiol Meas 2023; 44: 114001. 10.1088/1361-6579/ad0425 37848016

[65] Mazzucco S Li L Tuna MA , et al. Age-specific sex-differences in cerebral blood flow velocity in relation to haemoglobin levels. Eur Stroke J 2024; 9: 772–780. 38634499 10.1177/23969873241245631 PMC11343687

[66] Alwatban MR Aaron SE Kaufman CS , et al. Effects of age and sex on middle cerebral artery blood velocity and flow pulsatility index across the adult lifespan. J Appl Physiol Bethesda Md 1985 2021; 130: 1675–1683. 10.1152/japplphysiol.00926.2020 PMC8285608 33703940

[67] Davies A Gurung D Ladthavorlaphatt K , et al. The effect of CO2 on the age dependence of neurovascular coupling. J Appl Physiol Bethesda Md 1985 2024; 137: 445–459. 10.1152/japplphysiol.00695.2023 38961823

[68] Hosford PS Gourine AV. What is the key mediator of the neurovascular coupling response? Neurosci Biobehav Rev 2019; 96: 174–181. 30481531 10.1016/j.neubiorev.2018.11.011 PMC6331662

[69] Hosford PS Wells JA Nizari S , et al. CO2 signaling mediates neurovascular coupling in the cerebral cortex. Nat Commun 2022; 13: 2125. 35440557 10.1038/s41467-022-29622-9 PMC9019094

[70] Iadecola C Zhang F. Permissive and obligatory roles of NO in cerebrovascular responses to hypercapnia and acetylcholine. Am J Physiol 1996; 271: R990–1001. 8897992 10.1152/ajpregu.1996.271.4.R990

[71] Harper AM Glass HI. Effect of alterations in the arterial carbon dioxide tension on the blood flow through the cerebral cortex at normal and low arterial blood pressures. J Neurol Neurosurg Psychiatry 1965; 28: 449–452. 5838479 10.1136/jnnp.28.5.449 PMC495935

[72] Iwabuchi T Kutsuzawa T Ikeda K , et al. Effects of blood gases on the pressure-flow relationships in canine cerebral circulation. Stroke 1973; 4: 65–72. 4685810 10.1161/01.str.4.1.65

[73] Liu G Cull G Wang L , et al. Hypercapnia impairs vasoreactivity to changes in blood pressure and intraocular pressure in rat retina. Optom Vis Sci 2019; 96: 470–476. 31274734 10.1097/OPX.0000000000001400 PMC6730557

[74] Ito H Kanno I Ibaraki M , et al. Changes in human cerebral blood flow and cerebral blood volume during hypercapnia and hypocapnia measured by positron emission tomography. J Cereb Blood Flow Metab 2003; 23: 665–670. 12796714 10.1097/01.WCB.0000067721.64998.F5

[75] Friend AT Balanos GM Lucas SJE. Isolating the independent effects of hypoxia and hyperventilation-induced hypocapnia on cerebral haemodynamics and cognitive function. Exp Physiol 2019; 104: 1482–1493. 31342596 10.1113/EP087602

[76] Ladthavorlaphatt K Surti FB Beishon LC , et al. Depression of dynamic cerebral autoregulation during neural activation: the role of responders and non-responders. J Cereb Blood Flow Metab 2024; 44: 1231–1245. 38301726 10.1177/0271678X241229908 PMC11179612

[77] Panerai RB Davies A Clough RH , et al. The effect of hypercapnia on the directional sensitivity of dynamic cerebral autoregulation and the influence of age and sex. J Cereb Blood Flow Metab 2024; 44: 272–283. 37747437 10.1177/0271678X231203475 PMC10993882

[78] Smith KJ Ainslie PN. Experimental physiology regulation of cerebral blood flow and metabolism during exercise. Physiol Soc Exp Physiol 2017; 102: 1356–1371. 10.1113/EP086249 28786150

[79] Coverdale NS Gati JS Opalevych O , et al. Cerebral blood flow velocity underestimates cerebral blood flow during modest hypercapnia and hypocapnia. J Appl Physiol (1985) 2014; 117: 1090–1096. 25012027 10.1152/japplphysiol.00285.2014

[80] Verbree J Bronzwaer A-SGT Ghariq E , et al. Assessment of middle cerebral artery diameter during hypocapnia and hypercapnia in humans using ultra-high-field MRI. J Appl Physiol (1985) 2014; 117: 1084–1089. 25190741 10.1152/japplphysiol.00651.2014

